# A facile strategy of using MoS_2_ quantum dots for fluorescence-based targeted detection of nitrobenzene†‡

**DOI:** 10.1039/d3ra00912b

**Published:** 2023-05-12

**Authors:** Bhasha Sathyan, Ann Mary Tomy, Neema PM, Jobin Cyriac

**Affiliations:** a Department of Chemistry, Indian Institute of Space Science and Technology Thiruvananthapuram Kerala 695 547 India; b School of Physics, Indian Institute of Science Education and Research Thiruvananthapuram India jobincyriac@iist.ac.in

## Abstract

We present a simple approach for producing photoluminescent MoS_2_ quantum dots (QDs) using commercial MoS_2_ powder as a precursor along with NaOH and isopropanol. The synthesis method is particularly easy and environmentally friendly. The successful intercalation of Na^+^ ions into MoS_2_ layers and subsequent oxidative cutting reaction leads to the formation of luminescent MoS_2_ QDs. The present work, for the first time, shows the formation of MoS_2_ QDs without any additional energy source. The as-synthesized MoS_2_ QDs were characterized using microscopy and spectroscopy. The QDs have a few layer thicknesses and a narrow size distribution with an average diameter of ∼3.8 nm. Nitrobenzene (NB), an industrial chemical, is both toxic to human health and dangerously explosive. The present MoS_2_ QDs can be used as an effective photoluminescent probe, and a new turn-off sensor for NB detection. The selective quenching was operated *via* multiple mechanisms; electron transfer between the nitro group and MoS_2_ QDs through dynamic quenching and the primary inner filter effect (IFE). The quenching has a linear relationship with NB concentrations from 0.5 μM to 11 μM, with a calculated detection limit of 50 nM.

## Abstract 2

We present a simple approach for producing photoluminescent MoS_2_ quantum dots (QDs) using commercial MoS_2_ powder as a precursor along with NaOH and isopropanol.

## Graphical abstract

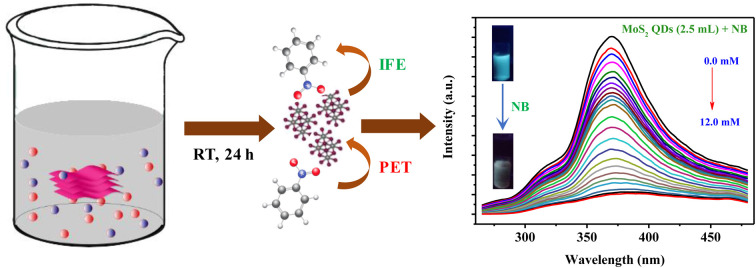

## Introduction

The advent of graphene triggered interest in the synthesis of morphologically similar 2D layered materials.^1–4^ Even though graphene exhibits unique properties, it appears to be inadequate for many electrical and optical applications due to its zero bandgap energy. Characteristic graphene analogous 2D structures, transition metal dichalcogenides (TMDs), have garnered increasing attention as a result of the significant advancements made *via* graphene.^5^

Molybdenum disulfide (MoS_2_), a TMD with a large intrinsic bandgap and excellent carrier mobility that resembles graphene, has created new opportunities for technological advancement. The framework of MoS_2_ comprises weak van der Waals-coupled layers of S–Mo–S, where a Mo atomic layer is sandwiched between two layers of S atoms. Therefore, we can readily exfoliate the layered skeleton of MoS_2_ to tune the bandgap ranging from an indirect (1.2 eV) for bulk material to a direct bandgap semiconductor (1.9 eV) for single-layer MoS_2_. Modified electronic properties due to the persistent adjustable bandgap in the MoS_2_ layer cause strong luminescence.^6–9^ The quantum confinement, edge effects, and photoluminescence in MoS_2_ nanomaterial has caused a great sensation in a variety of applications, including energy storage, electronics, thermoelectric, catalysis, gas sensing, biomedicine, and analytical sensing. Thus, researchers have paid attention to developing different synthetic strategies for preparing photoluminescent MoS_2_ nanostructures such as electrochemical etching,^10^ mechanical exfoliation,^11^ hydrothermal synthesis,^12^ chemical vapor deposition,^13,14^ lithium intercalation,^15^ electron-fenton reaction processing,^16^ sonication combined with solvothermal treatment synthesis,^17^ liquid exfoliation in organic solvents, *etc.* The liquid-phase exfoliation (LPE) in organic solvents are some of the most viable techniques for high yield fabrication of a few layers of MoS_2_ nanostructures. However, retrieving materials for use in other applications from liquid dispersions without layer aggregation is still hard. Hence, it is pivotal to modify liquid phase exfoliation for the effective extraction of the monolayer of MoS_2_ nanostructure.^18–22^

**Scheme 1:**
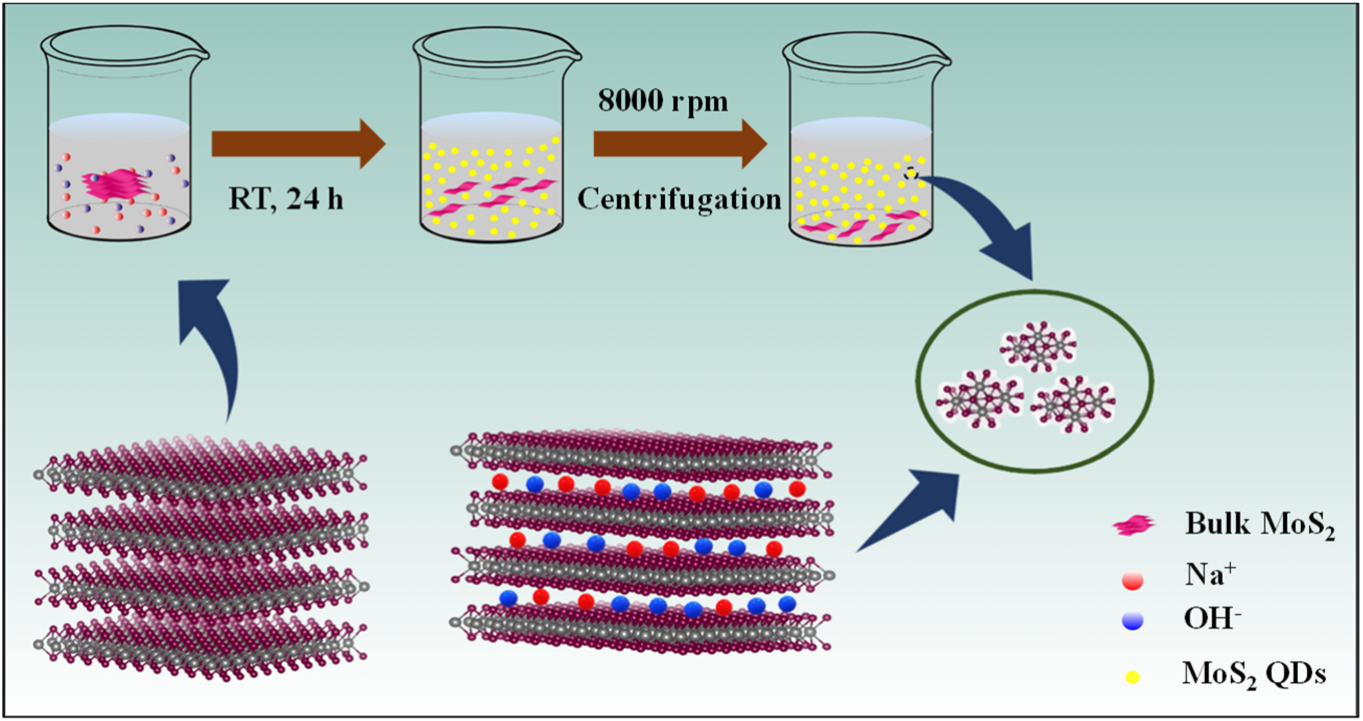
Formation of MoS_2_ QDs.

Herein, we introduce a simple and environmentally friendly route to prepare MoS_2_ quantum dots (QDs) by ion intercalation into the bulk material in a suitable solvent under optimized conditions without any external stimulation (Scheme 1). We have successfully exfoliated heterodimensional MoS_2_ QDs using NaOH in the presence of a polar volatile solvent, isopropyl alcohol (IPA), and contrive the MoS_2_ QDs for the label-free turn-off fluorescent sensor.

The Na^+^ and OH^−^ ions are intercalating into the layered bulk MoS_2_ to weaken the layer–layer interaction forming photoluminescent MoS_2_ QDs. The ionic radius of the intercalation reagent must be less than the interlamellar spacing of MoS_2_ (6.5 Å). The intercalation is driven using sodium ions (1.78 Å) and the hydroxyl group (2.68 Å),^23^ which have smaller ionic radii, as efficient “intercalation reagents”. The availability of IPA solution increases the intercalation efficiency of Na^+^ and OH^−^ ions for the quick and force-free synthesis of luminescent MoS_2_ QDs.

With the advent of global industrialization, a significant amount of nitroaromatic compounds and other harmful organic analytes are discarded into the environment, occasionally without screening. Hence, the development of sensors for the recognition and detection of the organic analyte has garnered tremendous interest among all sensors as it has a direct effect on one's health and environment, which jeopardizes the green economy.^24^ Nitrobenzene (NB), one of the most indispensable organic intermediates is used in the production of many chemicals, including aniline, *m*-phenylenediamine and trinitro toluene (TNT). However, it is non-biodegradable, highly toxic and sedimentable. The WHO International Agency for Research on Cancer has categorized NB as a carcinogen, making it a representative harmful substance for both humans and the environment. Therefore, efficient NB detection techniques are essential to lessen its negative effects on the environment and people. The NB has been detected by different methods which include ion mobility spectrometry (IMS),^25^ surface-enhanced Raman spectroscopy (SERS),^26^ gas chromatography coupled with mass spectrometry (GC-MS),^27^ and cyclic voltammetry.^28^ Nonetheless, these techniques are constrained by cost, pricey equipment, and challenging test procedures. Thus, it is important to keep an eye on a streamlined, low-cost, and accurate method for NB detection.^7,29–32^ Here, we employed MoS_2_ QDs fluorescent probe as an approach for the simple, selective, and sensitive detection of NB. The addition of micromolar concentration of NB causes the quenching of strong fluorescence of MoS_2_ QDs. It was encountered that the quenching mechanism works through a wide range of processes. Inquisition of the luminescence quenching mechanism of NB indicates the domination of the electron transfer due to the nitro group at the excited state and the presence of the inner-filter effect (IFE).

## Experimental section

### Materials and reagents

MoS_2_ powder was purchased from Sigma-Aldrich (USA). Isopropyl alcohol (IPA) and *N*-methyl-2-pyrrolidone (NMP) were purchased from Spectrochem, India. All the other reagents: sodium hydroxide (NaOH), nitrobenzene (NB), *N*,*N*-dimethylformamide (DMF), toluene, aniline, dimethylacetamide (DMA), acetonitrile (CH_3_CN), dichloromethane (CH_2_Cl_2_), chloroform (CHCl_3_), chlorobenzene (PhCl) and benzene were purchased from Merck (India). All chemicals were analytical grade and used without the additional purification. Deionized water was used in the experiments.

### Instrumentation

The high-resolution transmission electron microscope (TEM) images of MoS_2_ QDs were collected using a JEOL JEM 2100 instrument and dynamic light scattering (DLS) was performed by Zetasizer nano series (Malvern Panalytical). X-ray diffraction (XRD) studies were performed using a Bruker Discover D8 diffractometer equipped with Cu Kα radiation for elucidation of the crystal phase of the material. Raman spectra were collected using a Renishaw micro-Raman spectrometer with an excitation laser wavelength of 532 nm. The elemental and bonding configuration were characterized using X-ray photoelectron spectrophotometer (Kratos-Analytical Axis Ultra, XPS) equipped with a monochromatic Al Kα radiation source. Fourier transform infrared spectra (FTIR) were measured using a PerkinElmer Spectrum 100 FT-IR spectrometer. The surface morphology imaging of MoS_2_ QDs was performed using field emission scanning electron microscopy (Quanta FEG200, FEI, USA) with elemental mapping. A Cary 100 Bio UV spectrometer were used to record the UV-visible absorption spectrum. All the fluorescence study and lifetime measurements were carried out using Fluoromax-4 Spectrofluorometer with a slit width of 4 nm and integration time of 0.1 s.

### Synthesis of MoS_2_ QDs

The MoS_2_ QDs were synthesized by using commercial MoS_2_ powder and NaOH in a solution of IPA. In brief, 100 mg of MoS_2_ powder was mixed with 25 mg of NaOH in 10 mL of IPA. The mixture of these precursors was simply reserved for 24 h at room temperature. The intercalation reaction proceeds without any external stimulation. The MoS_2_ QDs formed were separated by centrifugation at a speed of 8000 rpm for 20 min to remove large particles followed by dialysis for 24 h to remove the excess NaOH using a 1000 Da MWCO (molecular weight cut-off) dialysis tube.

### Detection of NB using MoS_2_ QDs

The detection of NB by MoS_2_ QDs was performed as follows: the MoS_2_ QDs (2 mL, pH 13) and various concentration of NB (100 μL) solution were successively micropipetted into a 3.5 mL cuvette. The resulting mixture was properly blended and the photoluminescence emission spectrum of the solution were gathered after incubation for 1 min. The sensing study was conducted using an excitation wavelength of 250 nm.

## Results and discussion

### Characterization of the MoS_2_ QDs

**Fig. 1:**
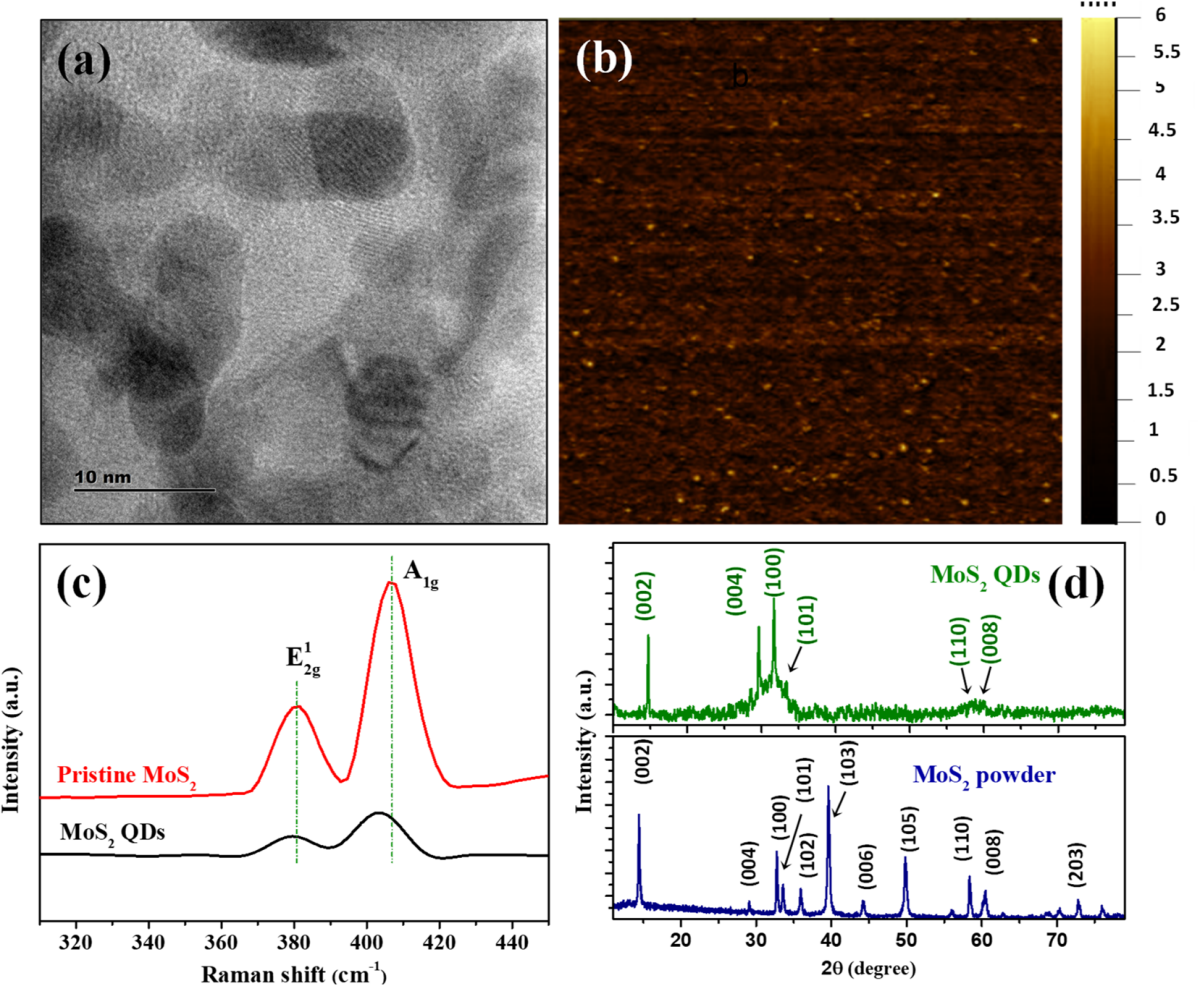
(a) TEM image of the MoS_2_ QDs. (b) AFM image of MoS_2_ QDs. (c) Raman spectra of pristine MoS_2_ powder (red trace) and MoS_2_ QDs (black trace). (d) XRD diffractograms of MoS_2_ QDs and MoS_2_ powder. The ICDD ref. no. used for MoS_2_ powder is 04-003-3374.

The MoS_2_ QDs were thoroughly characterized to unravel their morphology, photoluminescence properties, and chemical identities. A representative TEM image of MoS_2_ QDs given in Fig. 1a shows a near spherical morphology and an average diameter of ∼3.8 nm (calculated from >100 MoS_2_ QDs in different TEM images). The TEM images given in Fig. S1a&b‡ shows that the as synthesized MoS_2_ QDs have a *d*-spacing value of 0.26 nm which represent (100) planes of MoS_2_. Further, SEM image (see Fig. S1c(i)‡) and EDS measurements were performed to demonstrate morphology and constituent element. Elemental mapping was executed to obtain information about the distribution of constituent's elements of MoS_2_ QDs as shown in Fig. S1c(ii–iv).‡ Correspondingly, the hydrodynamic diameter and size distribution were measured using DLS is depicted in Fig. S1d‡ shows the average size of ∼4.4 nm, which is in close alliance with the TEM data.^33^ The height profile obtained from AFM analysis (given in Fig. S1e‡) shows an average height of ∼2 nm, which correspond to 2–3 layers.^34^

The number of layers of MoS_2_ QDs were examined by Raman spectroscopy, and the spectra are shown in Fig. 1c. Two distinct Raman peaks could be found at 380.6 cm^−1^ and 406.9 cm^−1^ (*Δ* = 26.3 cm^−1^) for pristine MoS_2_. These peaks were attributed to the in-plane (E_2g_^1^) and vertical plane (A_1g_) vibration of the Mo–S bond in 2H MoS_2_.^35^ The respective bands for MoS_2_ QDs nanostructure were observed at 380.4 cm^−1^ and 403.3 cm^−1^ and the frequency difference was *Δ* = 22.9 cm^−1^. It was apparent that the MoS_2_ QDs had a change in Raman shift values in the A_1g_ mode compared to bulk counterparts, which was owed to the fact that as layer thickness was decreased thereby E_2g_^1^ become stiffer and A_1g_ happen to softer.^36,37^ The frequency difference between the E_2g_^1^ and A_1g_ mode of vibration assert the formation of MoS_2_ QDs with ∼3 layers.^38^ The crystalline phase of MoS_2_ QDs was identified by XRD diffractogram (Fig. 1d). The peaks were assigned to (002), (004), (100), (101), 110 and (112) planes of MoS_2_, respectively.^39^ The peak positions are matching with the ICDD ref. no. 04-003-3374. The well-defined peaks in the XRD pattern affirm the presence of 2H phase of MoS_2_ QDs with good crystallinity. According to the previous report, the peak broadening in the diffraction pattern and appearance of less number of peaks compared to that of MoS_2_ powder is attributed to the formation of a few layer containing MoS_2_ nanoparticles with weaker interlayer interaction.^9,35,40^

**Fig. 2:**
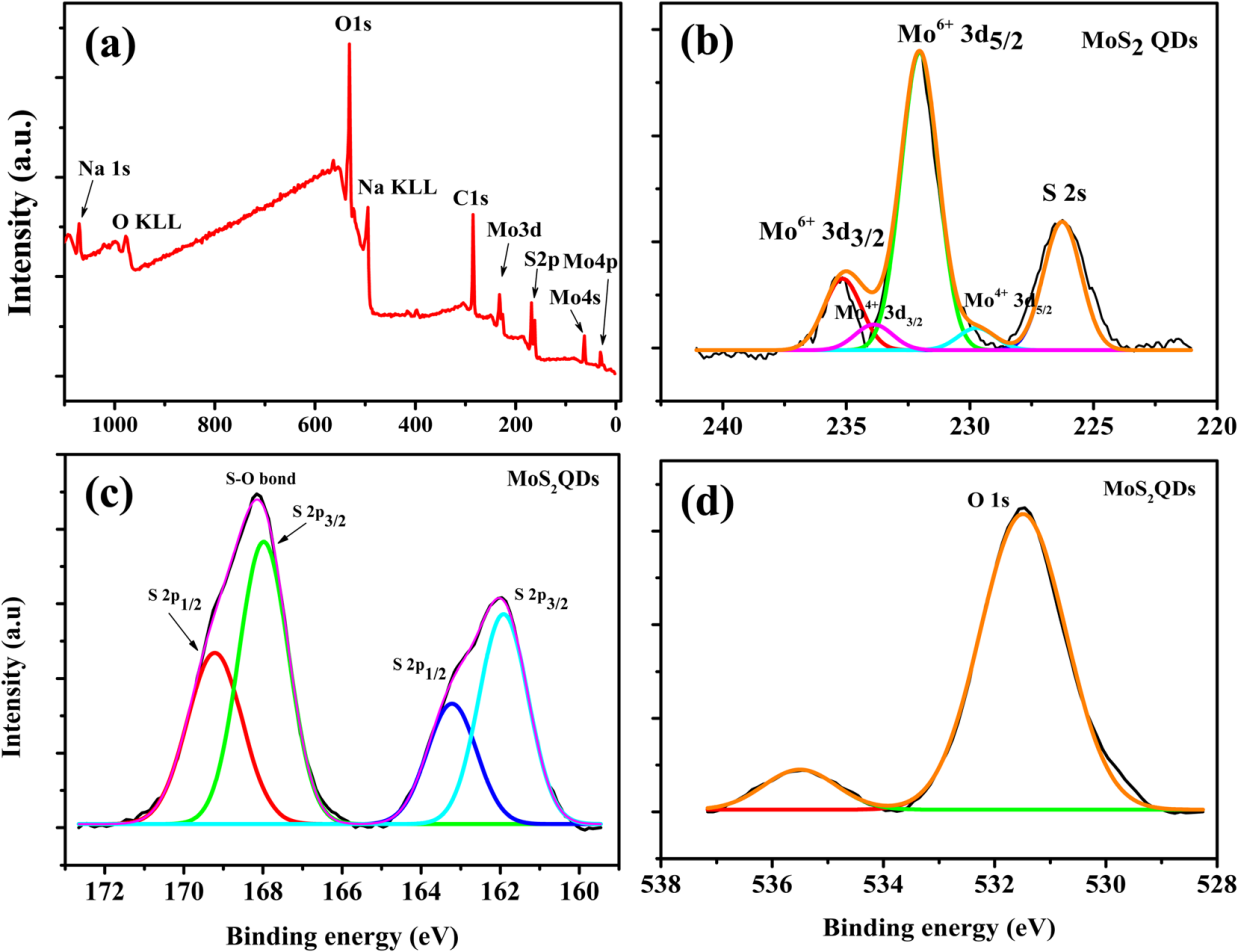
(a) The survey scan XPS spectrum of the MoS_2_ QDs (b), (c) and (d) are the high resolution XPS spectra of Mo 3d, S 2p and O 1s peaks, respectively.

To understand the chemical composition and phase state of MoS_2_ QDs, X-ray photoelectron spectroscopy (XPS) were employed. Peaks corresponding to Mo 3d, S 2p, C 1s and O 1s peaks were observed in the survey spectrum (Fig. 2a). For Mo, the peak centered at 232.6 eV and 229.7 eV correspond to Mo 3d_3/2_ and Mo 3d_5/2_ of Mo^4+^, while two orbital splitting peak of Mo 3d at 235.2 eV (Mo 3d_3/2_) and 232.1 eV (Mo 3d_5/2_) were assigned to Mo^6+^ (Fig. 2b).^41^ The Mo^6+^ is ascribed to the oxidation of Mo edges in the QDS. The intense Mo^6+^ peak indicates that the Mo^4+^ largely underwent oxidation during the formation of QDs. In addition, the peak at 226.3 eV is attributed to S 2s peak. The S^2−^ doublets is observed at 162 eV and 163.3 eV (Fig. 2c). Further, a higher binding energy peak is present at 168 eV and 169.2 eV corresponds to the prescence of an S–O bond.^5^ In addition, the presence of O 1s peak also confirmed the oxidation of MoS_2_ QDs.^42^ In the high resolution spectra of O 1s (Fig. 2d), the peak at 531.5 eV was due to O^2−^ and the peak at 534.4 eV was assigned to the O of NaOH.^42^

**Fig. 3:**
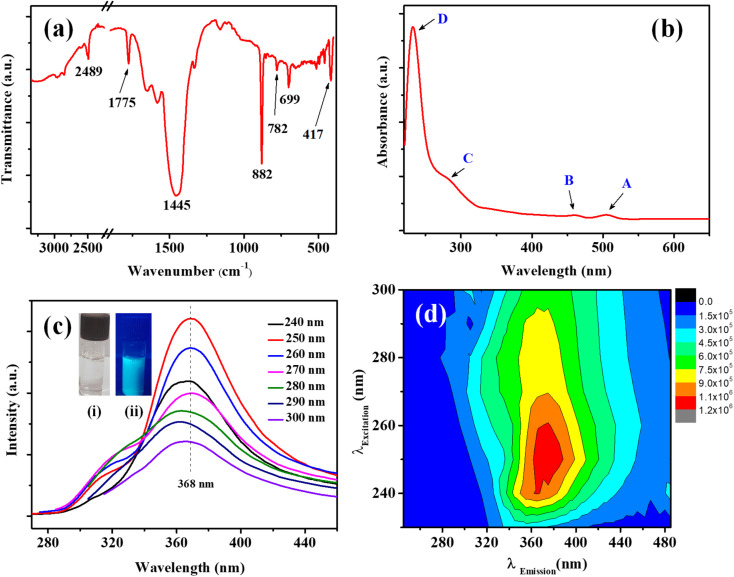
(a) FTIR spectrum of MoS_2_ QDs. (b) UV-visible absorption spectrum of the MoS_2_ QDs, (c) emission spectra of MoS_2_ QDs at excitation wavelength progressively increasing from 240 to 300 nm. Inset photographs given in (c) are MoS_2_ QDs under visible light (i) and UV light at 365 nm (ii). (d) A contour map of the three-dimensional fluorescence spectra of MoS_2_ QDs.

In the FTIR spectrum (Fig. 3a) the characteristic Mo–S bonds is appeared at 417 cm^−1^.^43^ The other important infrared peaks observed at 699, 782, and 882 cm^−1^ were attributed to sulfate vibrations.^44^ The UV-visible absorption and photoluminescence (PL) characteristics of the MoS_2_ QDs were examined. Lateral attributes of the exfoliated nanostructures were evident in the UV-visible absorption spectrum (Fig. 3b). The UV-visible spectrum given in Fig. 3b shows absorption peaks at 231 (D), 278 (C), 460 (B), and 505 (A) nm which are the characteristic peak of exfoliated MoS_2_ QDs. The peak at 505 nm (A) and 460 nm (B) is attributed to the direct transition from the deep valence band to the conduction band.^45,46^ Two high energy absorption peaks near the UV region at 231 nm (D) and 278 nm (C) can be assigned to the excitonic features of MoS_2_ QDs.^47^ A detailed fluorescence study was carried out by exciting MoS_2_ QDs at different excitation wavelength. It was noted that the best emission of MoS_2_ QDs was at 368 nm upon exciting at 250 nm (Fig. 3c). The photoluminescence spectra of the QDs shows an excitation-dependent fluorescence depicts the polydispersity of MoS_2_ QDs which concur with the earlier reports.^39,48,49^ The fluorescent quantum yield, calculated using quinine sulfate as a reference, was 4.4%, comparable to those MoS_2_ QDs reported previously.^49^ Excitation and emission contour map MoS_2_ QDs (Fig. 3d) depicted multicolor emission.

To employ in practical applications, the PL response of fluorophores must be consistent and stable. Thus, the stability of fluorescent MoS_2_ QDs was tested by measuring the emission intensity of MoS_2_ QDs over a period of 30 days. The subsequent spectral data are portrayed in Fig. S2.‡ The emission intensity was not changed up to 30 days. These findings demonstrate that the MoS_2_ QDs are very stable in suspension medium even after 30 days. Further, the photostability of MoS_2_ QDs was tested under the constant irradiation of MoS_2_ QDs with UV light for 120 minutes and the results are shown in Fig. S3.‡ As depicted in Fig. S3‡ no discernible changes in the emission intensity were observed, demonstrating the excellent photostability of MoS_2_ QDs, making it a rightful candidate for applications such as fluorescence imaging.

### Photoluminescence quenching characteristic of MoS_2_ QDs toward NB

**Fig. 4:**
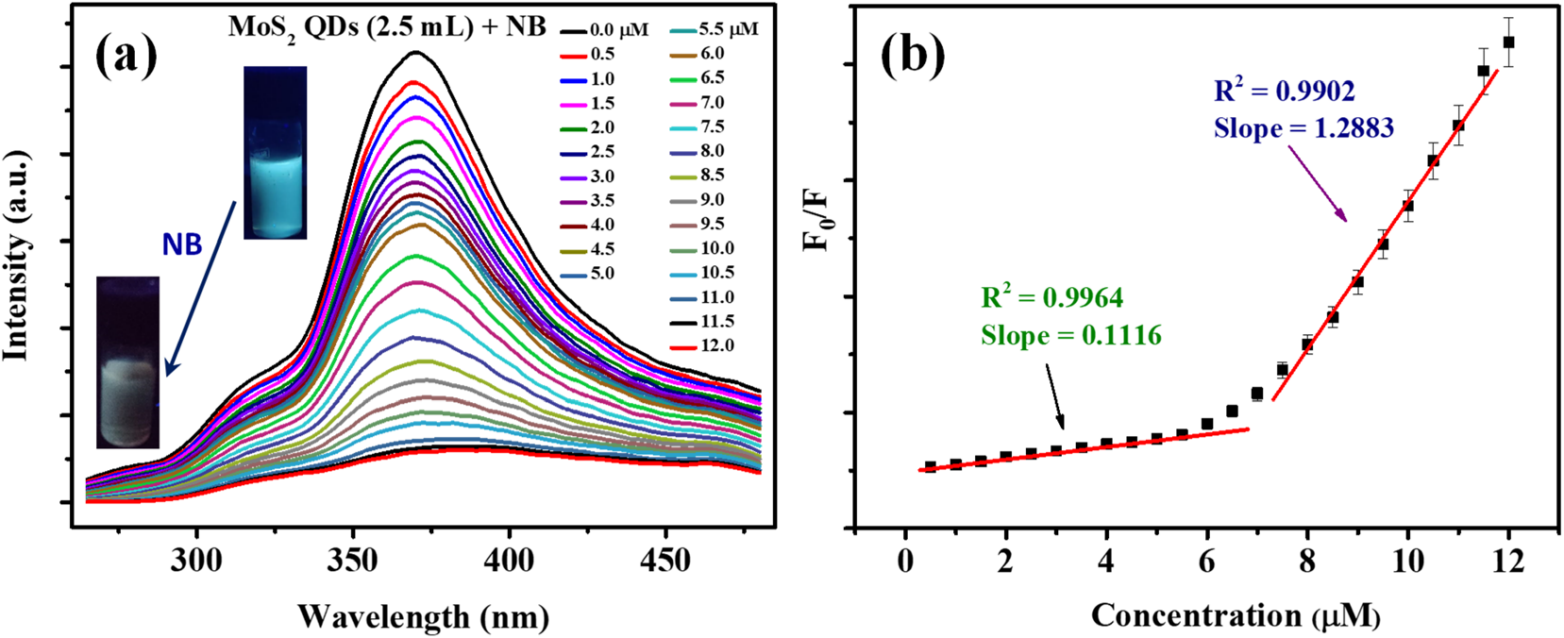
(a) Fluorescence response of MoS_2_ QDs in the presence of various concentrations of NB. The concentrations of NB are given in figure legend. The inset shows the photographs of MoS_2_ QDs sensor solution before and after the addition of NB. The photograph was taken under UV light (365 nm, 8 W) (b) showing the dynamic range of MoS_2_ QDs sensor towards NB detection. *F*_0_ and *F* are the fluorescence intensity of MoS_2_ QDs before and after the addition of NB, respectively.

Fluorescent nanomaterials have been used in the sensitive detection of variety of chemical species including nitroaromatic explosives.^50–52^ The present MoS_2_ QDs with excellent photoluminescence characteristics were used as an effectual fluorescent probe for NB detection and the work is representing one of the first reports of NB detection using MoS_2_ QDs. Fig. 4a delineates the spectrofluorimetric response of MoS_2_ QDs with the addition of NB. It is clear that the NB causes a monotonous decrease in the PL of MoS_2_ QDs and the response was found to be linear. As seen in the photograph given in the inset of Fig. 4a, the MoS_2_ QDs solution has a strong blue emission when exposed to a 365 nm UV lamp (8 W), where's upon addition of NB, the photoluminescence was no longer visible to the naked eye, asserting that the fluorescence had been quenched.

From Fig. 4b, one can observe that the system can react to the presence of NB concentration as low as 0.5 μM. The calibration curve shows two linear ranges: concentrations from 0.5 μM to 6 μM and from 6 μM to 11 μM. The reason attributed to the presence of two linear ranges is explained later in the text. The fluorescence quenching of MoS_2_ QDs is consistent with a standard Stern–Volmer type equation.

1 *F*_0_/*F* = 1 + *K*_sv_ [NB]

where, *F*_0_ is the PL intensity at a specified emission wavelength in the absence of NB, *F* denotes the PL intensity in the presence of NB and *K*_SV_ represents the Stern–Volmer quenching constant.^53^ The *K*_SV_ value for the first linear range (0.5 μM to 6 μM) was 0.1116 × 10^6^ M^−1^ (*R*^2^ = 0.9964) and for the linear range from 6 μM to 11 μM it was 1.288 × 10^6^ M^−1^ (*R*^2^ = 0.9972). The limit of detection (LOD) was estimated to be 50 nM (LOD = 3*s*/*b*, where *s* is the standard deviation of the blank and *b* is the slop of the calibration). We compared the present sensor with the literature. Table S1‡ summarizes different nitrobenzene sensors from literature, their detection limit, dynamic range, selectivity, and the sensor mechanism. From the table one can conclude that performance of present NB sensor is comparable to the best reported values.^32^ Similarly, PL quenching of MoS_2_ QDs solution with different concentrations of Hg^2+^, NMP, PhCl, and DMF was performed. The emission spectra were collected for each concentration of four quenchers and the Stern–Volmer equation was fitted for each set of data obtained. Table 1 lists the parameters for each quencher connected to the fitted Stern–Volmer model. The order of Stern–Volmer quenching constant for the five quenchers was NB >> Hg^2+^ > NMP > PhCl > DMF. Here, NB displayed superior PL quenching and the highest *K*_SV_ value (1.288). The ratio of *K*_SV_ of NB and the other four quenchers determined the selectivity factor (SF), and the SF value was applied to assess the selectivity of MoS_2_ QDs toward NB detection. From Table 1, the SF value for, Hg^2+^, NMP, PhCl, and DMF are 0.0609, 0.0274, 0.0163, and 0.0098, respectively indicating that the PL response of MoS_2_ QDs are altered significantly towards the presence of NB than the other four quenchers.

**Table 1:** Comparison of *K*_sv_ values for different analytes, showing the best *K*_sv_ for NB

System	*K*_sv_ [×10^6^ M^−1^]	*R* ^2^	SF
NB	1.2883	0.9972	1.00
Hg^2+^	0.0785	0.9958	0.0609
NMP	0.0353	0.9953	0.0274
PhCl	0.0210	0.9948	0.0163
DMF	0.0126	0.9923	0.0098

### The quenching mechanism

Fluorescence quenching can occur for a variety of causes. Based on the literature available on fluorescence NB sensing, it has been suggested that the sensors primarily operate on three possible mechanisms. These are (a) photo-induced electron transfer (PET), (b) inner filter effect (IFE) or/and Föster resonance energy transfer (FRET) and (c) ground state complex formation.^30,32,54^ It is prominent that NB is highly electron deficient in nature due to the presence of electron-withdrawing nitro group, whereas the zeta potential studies unravel that the MoS_2_ QDs are negatively charged (−39.4 mV). This results in the binding of the electron-deficient nitro group to the electron-rich MoS_2_ QDs which causes the quenching of fluorescence.^29,31,55,56^ The foregoing circumstance is appropriate for PET.

**Fig. 5:**
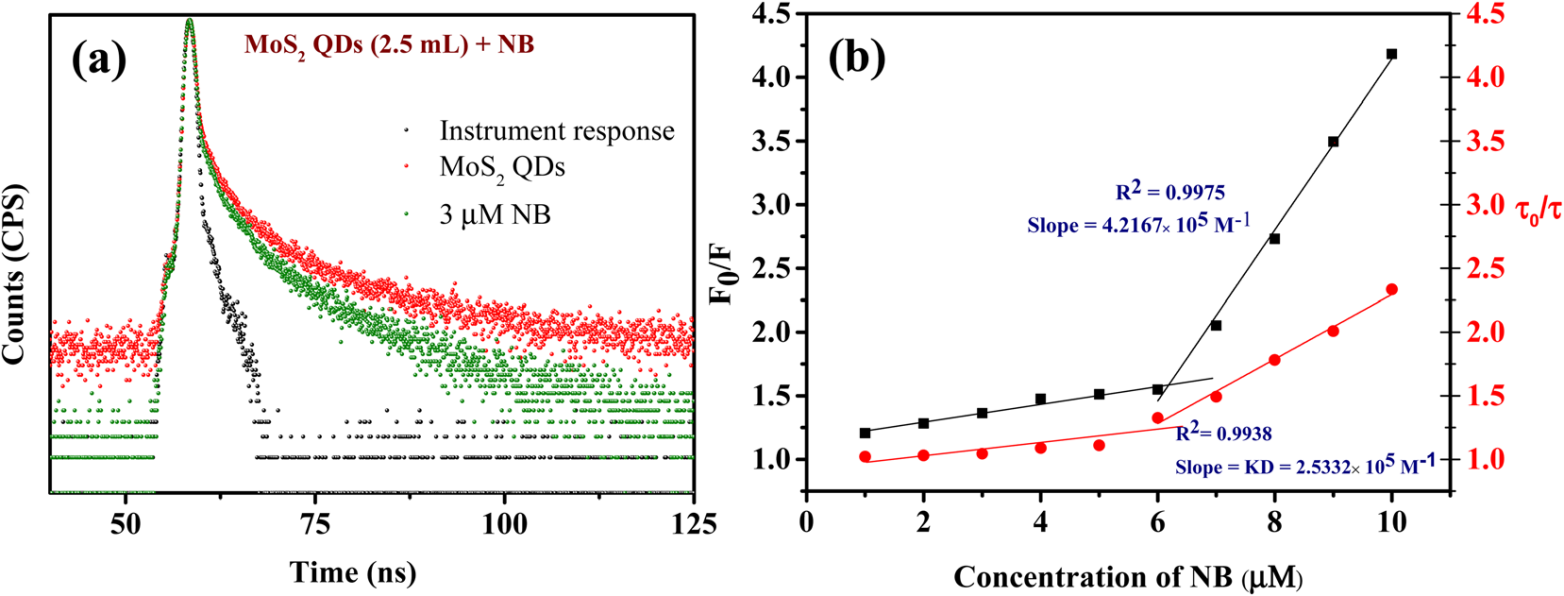
(a) Lifetime decay curves of MoS_2_ QDs (red) and MoS_2_ QDs with 3 μM NB (green). (b) Relationship between *τ*_0_/*τ* and *F*_0_/*F vs.* [NB], showing linear plot with different slopes. *F*_0_ and *F* are the steady state intensity before and after the addition of NB, respectively.

Initially, we should classify whether quenching process is static or dynamic in manner. Therefore, the fluorescence decay time of MoS_2_ QDs was examined with and without the presence of NB. All the lifetime decay profiles were fitted using triexponential functions, with a fast, moderate and a longest component, indicating the presence of various energy levels possible. It is interesting to note that there was a noteworthy decrease in the fluorescence lifetime of all the components of MoS_2_ QDs after the addition of NB (Fig. 5a and Table S2‡). The longest lifetime component of MoS_2_ QDs was found to be decreased from 19.46 ns to 11.05 ns and change in medium component was changed from 6.13 ns to 3.46 ns with a corresponding decrease in the contribution (47 to 26% and 32 to 26%, respectively). The decrease of fastest component, on the other hand was significant (1.78 ns to instrument response limited component of 0.45 ns), as it is accompanied by a huge increase in the contribution; from 19 to 66%. This increase in the non-radiative component is a direct evidence of excited state PL quenching of MoS_2_ QDs, due to the emergence of non-radiative excited state electron transfer between fluorophore and quencher (NB). Further, the incremental decrease of lifetime with respect to the various concentrations of analyte is an indication of presence of multiple quenching mechanisms. Thus, we can confer the presence of dynamic quenching.

In a similar fashion, fluorescence lifetime decay of MoS_2_ QDs with Hg^2+^, NMP, PhCl, and DMF was also analyzed and observed that there is no decrease in the lifetime of MoS_2_ QDs excluding any dynamic quenching. (Fig. S4(a–d)‡). This could be one of the reasons for the enhanced selectivity of the probe (MoS_2_ QDs) towards NB.

The involvement of static quenching is investigated using UV-vis absorption spectroscopy, as the presence of new complexes can undeniably be substantiated by the appearance of new absorption peaks. The absorption spectra of MoS_2_ QDs at various NB concentrations as shown in Fig. S5,‡ which shows an increment in the absorption intensity without causing any shift in the peak of MoS_2_ QDs. Hence we can presume that no ground state charge–transfer complex formation is occurred between MoS_2_ QDs and NB.^24^

For any molecular species in solution, the raise in temperature causes increased molecular collision. This can invariably weaken the noncovalent forces, if any, that contribute to the formation of complexes. Therefore, in the case of static quenching, the value of Stern–Volmer quenching constant (*K*_sv_) can decreases as temperature increases, due to the disruption of newly formed complex. While *K*_sv_ increases in the case of dynamic quenching, as it can facilitate the interactions of probe and the quencher. In order to corroborate the results obtained from time resolved fluorescence spectra, the fluorescence quenching titration were done at different temperatures (293 K, 303 K, 313 K and 323 K) to conclude the type quenching mechanism responsible for NB induced quenching of MoS_2_ QDs.^57^ The Stern–Volmer plots (Fig. S6‡) were created from the titration data acquired at four different temperatures after it was analyzed by eqn (1). From Fig. S6,‡ it can be inferred that slope of the linear Stern–Volmer plot (*K*_sv_) increases with raise in temperature, which indicates that quenching of MoS_2_ QDs' fluorescence by NB is due to dynamic rather than any complex formation.

Additionally, a relative quantification of the extent of dynamic quenching is performed by analyzing lifetime values. From Fig. 5b the plot of *τ*_0_/*τ versus* [Q] where *τ*_0_ and *τ* are the MoS_2_ QDs decay time in the absence and presence of quencher, respectively, indicates from 1 μM to 6 μM there is only a minor change in the fluorescence decay lifetime but from 6 μM onwards significant lifetime decay is observed with the quenching. Thus, we can see that the contribution of dynamic quenching increases with the concentration of NB. This endorse the possibility of existence of multiple fluorescence quenching mechanisms. The linear fitting of the plot (*τ*_0_/*τ vs.* [NB]) from 6 μM to 10 μM form an equation of straight line with the intercept of 1.1, and the slope of this equation represent the dynamic quenching constant (*K*_D_),^58^ which was 2.53 × 10^5^ M^−1^. This value of *K*_D_ defines the fluorescence quenching efficiency due to dynamic quenching mechanism.

**Fig. 6:**
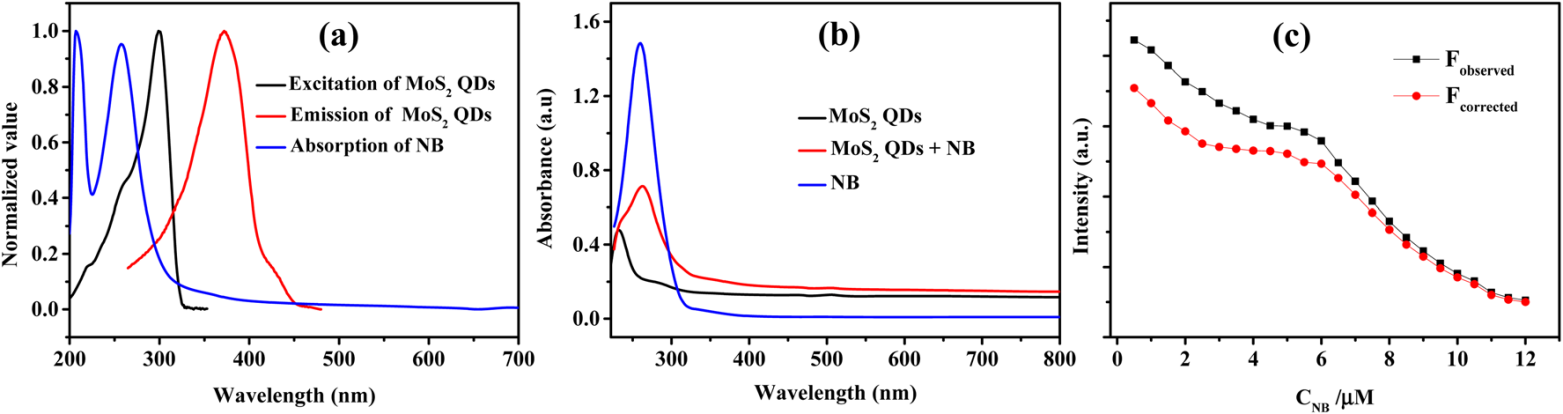
(a) Plot showing the spectral overlap between the absorption spectra of NB and excitation and emission spectra of MoS_2_ QDs. (b) Comparison of absorption response of MoS_2_ QDs, MoS_2_ QDs – NB system and NB. (c) Observed (black curve) and IFE corrected (red curve) fluorescence intensities of the sensor in the presence of NB.

To identify the other possible mechanisms influencing the quenching process, we concentrated on the resonance energy transfer processes such as Förster resonance energy transfer (FRET) and inner filter effect (IFE). Owing to the absence of spectral overlap of absorption spectra of NB and emission spectra of MoS_2_ QDs; one of the prior conditions for FRET to materialize, we can preclude the possibility of FRET. Hence the secondary inner-filter effect, FRET, is excluded from the absence of discernible overlap between emission spectra of MoS_2_ QDs and NB (Fig. 6a). Yet, the prospect for primary IFE is still feasible. In primary inner-filter effect, the fluorophore and quencher have a similar absorption range hence the energy provided for excitation of the fluorophore gets absorbed by the quencher.^59^ According to Fig. 6b, it is clear that the absorption spectra of MoS_2_ QDs overlap with the absorption spectra of NB. This observation lead us to conclude that the fluorescence quenching of MoS_2_ QDs by NB is due to dynamic quenching^60^ as well as primary inner-filter effect. To distinguish the involvement of excited state phenomena like dynamic quenching from the inner-filter effect, we determined the dynamic quenching efficiency from the steady state and time-resolved spectral studies. The fluorescence quenching efficiency was calculated as 79% using the fluorescence intensity of MoS_2_ QDs and MoS_2_ QDs-NB system (see eqn (1) and eqn (2) in ESI‡). This value of quenching efficiency was assigned to both the quenching mechanisms involved. The dynamic quenching efficiency component calculated using the average lifetime of MoS_2_ QDs in the absence and presence of NB was 47.3%. Since the inner-filter effect is a non-excited state phenomenon, the difference between the total quenching efficiency and dynamic quenching efficiency is likely the contribution solely from IFE (31.7%). IFE on the fluorescence quenching was further corrected based on the following formula^53^

2 *F*_corr(λex)_ = *F*_obs(λex)_/W

where, *W* is the correction factor that can be obtained from the equation.

3 *W* = (1–10^−*A*_FL_^)/*A*_FL_

**Scheme 2:**
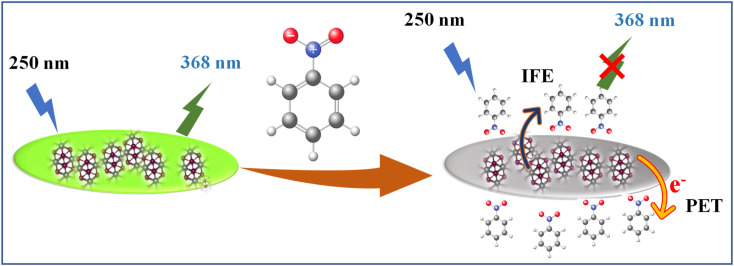
Illustration of sensing mechanism for NB using MoS_2_ QDs as fluorescent probe.

*F*_corr(λex)_ is the corrected fluorescence in the absence of IFE, *F*_obs(λex)_ is the measured fluorescence intensity and *A*_FL_ is the absorbance of the fluorescent component.^61^Fig. 6c illustrated that contribution of the quenching effect derive from the IFE decreases with the quencher concentration. Based on the observation, we intended that there was an excited state electron transfer interaction between the NO_2_ group of NB and electron-rich MoS_2_ QDs. Similarly, we depict the plot of the Stern–Volmer equation before and after correcting for the inner filter effect (IFE) as shown in Fig. S7.‡ The variation in graph depicts presence of multiple quenching mechanisms, which are PET and IFE in the present case (Scheme 2). Therefore, dynamic quenching and favorable IFE can explain the astounding selectivity of MoS_2_ QDs toward the NB.

### Selectivity studies

**Fig. 7:**
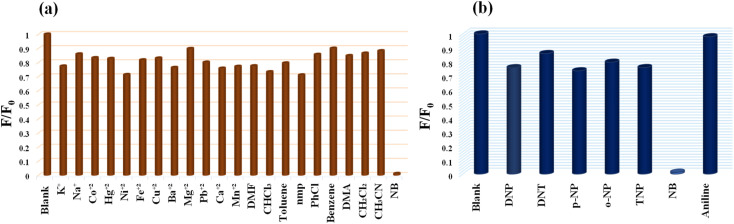
(a) Selectivity profile of MoS_2_ QDs toward NB over different metal ions and common organic solvents molecules. (b) Selectivity of MoS_2_ QDs for NB over other nitroexplosives.

The detection of NB in a competitive environment is highly desirable for real application purposes. To demonstrate this the selectivity of the MoS_2_ QDs-based photoluminescence sensor for NB *vs.* other chemicals (includes 12 metal ions, and 10 organic molecules) was examined by adding 100 μL of 1 mM solution of various analytes, and only NB caused a perceptible decrease in the photoluminescence intensity (Fig. 7a).

As shown in the Fig. S8a,‡ the luminescence intensity of MoS_2_ QDs at different analytes systems exhibit an extremely significant quenching effect on the addition of NB. Even though the development of efficient sensor platform for the selective NB detection has always been huge challenge, due to the interference in its detection caused by the other nitroexplosives. Interestingly, we noticed that the quenching percentage obtained for NB is 99.24% which is much higher than that obtained for paranitrophenol (*p*-NP, 55.78%), 2,4,6-trinitrophenol (TNP, 55.72%), *ortho* nitrophenol (*o*-NP, 22.28%) and 2,4-dinitrotoluene (DNT, 14.5%) (Fig. 7b). Further, *p*-NP (5 μM) was mixed with the MoS_2_ QDs, so that it could interact with MoS_2_ QDs prior to the additional mixing of NB (5 μM). It was noticed that addition of *p*-NP to the system cause no significant effect on the PL intensity, while NB (5 μM) caused considerable quenching of fluorescence even in the presence of *p*-NP. Similarly, competitive studies have done with the other nitroexplosives, and identical results were acquired. Showing that MoS_2_ QDs sensing probe have high selectivity towards NB even in the presence of interfering competitors (Fig. S8b‡). To support this fact, we have done the fluorescence lifetime decay analysis of MoS_2_ QDs with the interfering nitroexplosives (*p*-NP, TNP, *o*-NP and DNT). The insignificant change in the lifetime decay (Fig. S9(a–d)‡ of MoS_2_ QDs in presence of these nitroexplosives suggest that no excited state energy transfer is occurring. We attribute the selectivity of NB over other nitro explosives to size difference and presence intramolecular hydrogen bonding in the later. For example, the nitrophenol has both hydroxyl (–OH) group and a nitro group (–NO_2_) attached to the phenyl ring, while NB has only a nitro group attached to the benzene ring. The presence of the hydroxyl group in the nitrophenol allows the formation of intramolecular hydrogen bonds. Hence, the intramolecular hydrogen bonding within the molecule, prevent the contact of these analytes and MoS_2_ QDs sensor system.^62–64^ The reproducibility of the sensor was checked using two more batches of MoS_2_ QDs sensor. The synthesis of the MoS_2_ QDs, purification, the volume of the sensor solution, fluorescence measurement procedure, *etc.* has been kept the same for all batches. The data shows that the sensor material shows acceptable reproducibility. The data obtained from three batches are given in Fig. S10,‡ which is within 3 standard deviations of the data obtained for Fig. 4b. The high selectivity, excellent sensitivity and the reproducibility of the present sensor demonstrate a simple yet efficient platform for the selective detection of NB even in a competing environment.

## Conclusion

In conclusion, we have introduced a simple and cost-effective platform for the synthesis of MoS_2_ QDs by the ion intercalation method without any external stimuli. The intercalation of Na^+^ ions into MoS_2_ layers and subsequent oxidative cutting reaction leads to the formation of luminescent MoS_2_ QDs. The QDs were thoroughly characterized and has a 2H phase with a few layers thickness and an average diameter of ∼3.8 nm. The resultant QDs was used for the selective detection of NB. The NB sensor have good linear range and high sensitivity toward NB over other polar organic solvents and various metal ions. The excellent signal response for NB was enabled by dynamic quenching *via* electron transfer and primary inner-filter effect. According to our results, the same technique can be applied to other layered material, which opens the door to large scale easy synthesis of nanomaterial and can be used as a fluorescent probe in various applications.

## Conflicts of interest

There are no conflicts to declare.

## Supplementary Material

RA-013-D3RA00912B-s001
