# Supplementary material for: A facile strategy of using MoS2 quantum dots for fluorescence-based targeted detection of nitrobenzene
Source: RSC Adv. 2023 May 12;13(21):14614–24. doi: 10.1039/d3ra00912b (PMC10177963; doi:10.1039/d3ra00912b)
Supplement: RA-013-D3RA00912B-s001 [file RA-013-D3RA00912B-s001.pdf]

Electronic Supplementary Material (ESI) for RSC Advances.
This journal is © The Royal Society of Chemistry 2023

Electronic Supplementary Information

A facile strategy of MoS2 quantum dots for fluorescence-based targeted detection of nitrobenzene

Bhasha Sathyan,[a] Ann Mary Tomy,[a] Neema PM,[a,b] Jobin Cyriac[a]*
[a]Department of Chemistry, Indian Institute of Space Science and Technology, Thiruvananthapuram
[b]School of Physics, Indian Institute of Science Education and Research, Thiruvananthapuram
*Email: jobincyriac@iist.ac.in

Figure No.	Content	Page No.
S1	(a-b) The higher magnification TEM image of MoS_2 QDs. (c) EDS spectrum of MoS_2 QDs taken from SEM image (i) and EDS mapping of MoS_2 QDs (ii-iv) showing the elemental distribution. (d) The particle size distribution of MoS_2 QDs obtained from the dynamic light scattering (DLS) analysis. (e) Showing the height profile drawn on the AFM image.	2
S2	Fluorescence response of MoS_2 QDs for a period of 30 days	3
S3	Emission of MoS_2 QDs after exposure to UV irradiation (48 W power) at various time intervals	3
S4	Lifetime decay curve of MoS_2 QDs and MoS_2 QDs with different analytes such as (a) $HgCl_2$, (b) NMP, (c) PhCl, and (d) NMP.	4
S5	UV-visible spectra of MoS_2 QDs and MoS_2 QDs at different concentrations of NB (2.5µM-50 µM).	4
S6	Plots of F_0/F vs [Nitrobenzene] at different temperatures	5
S7	The stern-Volmer plot of fluorescence intensity before and after correcting the inner-filter effect	5
S8	(a) Percentage of quenching by different interfering analytes (0.01mM) before and after the addition of (0.01mM) NB. (b) Percentage of quenching by different interfering nitro explosives (0.01mM) before and after the addition of (0.01mM) NB.	6
S9	Lifetime decay curve of MoS_2 QDs and MoS_2 QDs with different nitro explosives such as (a) p-NP, (b) TNP, (c) o-NP, and (d) DNT	6
S10	The reproducibility of the sensor material	7
TS1	A comparison study with previously reported sensors	7
TS2	The lifetime component of MoS_2 QDs and MoS_2 QDs-NB	8

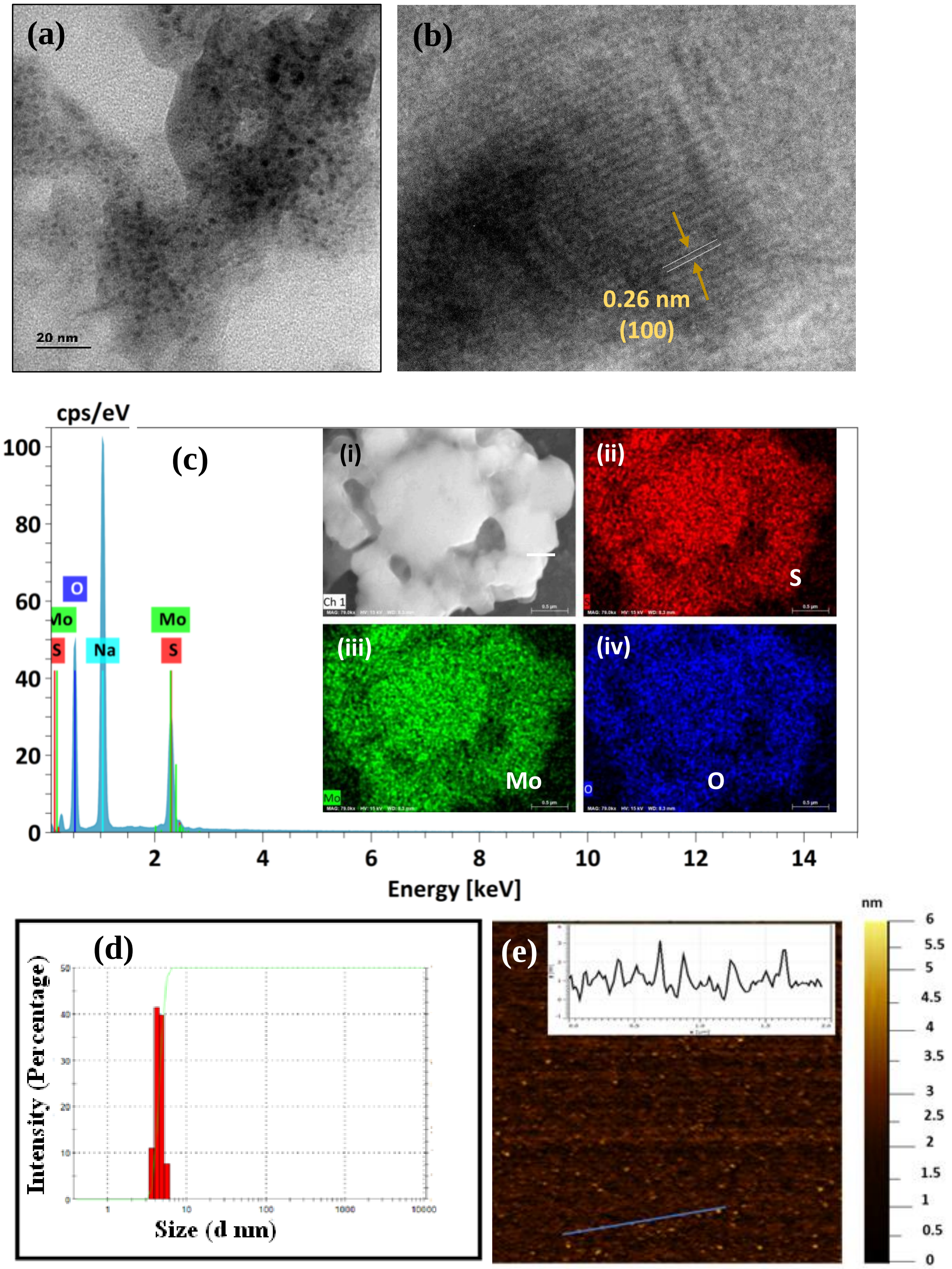

Fig. S1.(a) TEM micrograph of MoS_2 QDs. Scale bar is 20 nm. (b) A higher magnification image showing (100) planes (arrows indicating lattice fringe spacing corresponds to (100) plane). (c) EDS spectrum of MoS_2 QDs taken from the area shown in SEM image of MoS_2 QDs (i) and EDS mapping of MoS_2 QDs (ii-iv) showing the elemental distribution. (d) The particle size distribution of MoS_2 QDs obtained from the dynamic light scattering (DLS) analysis. (e) Showing the height profile drawn on the AFM image.

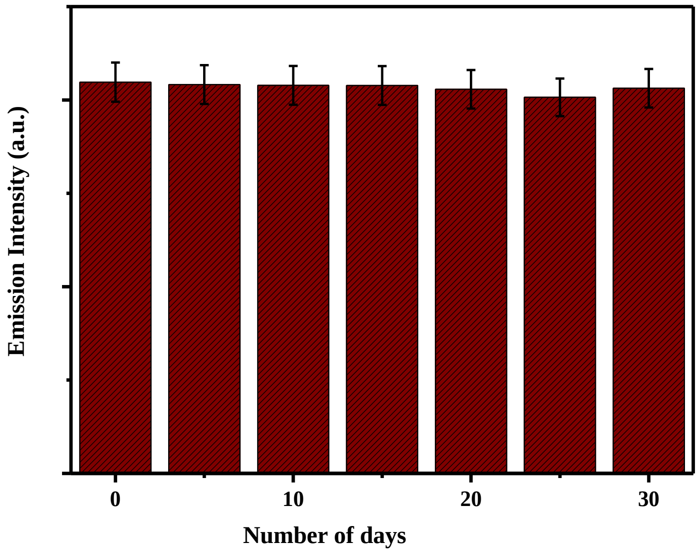

Fig. S2. Fluorescence response of MoS_2 QDs for a period of 30 days. The sample was kept under ambient conditions.

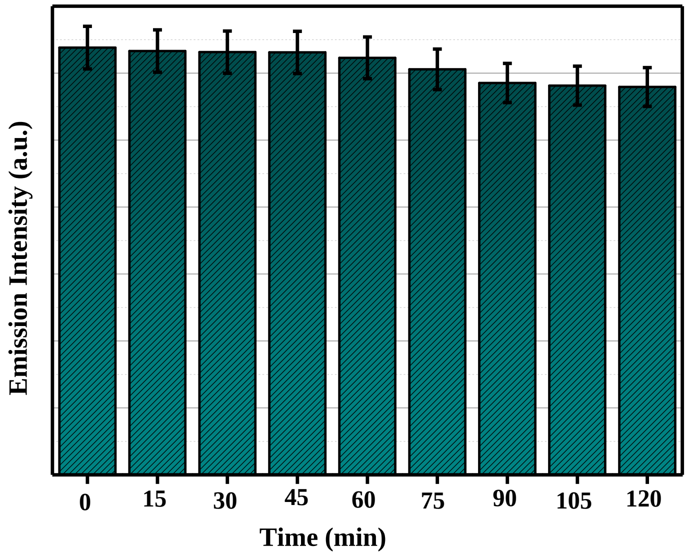

Fig. S3. Emission of MoS_2 QDs after exposure of UV irradiation (48 W power) at various time intervals.

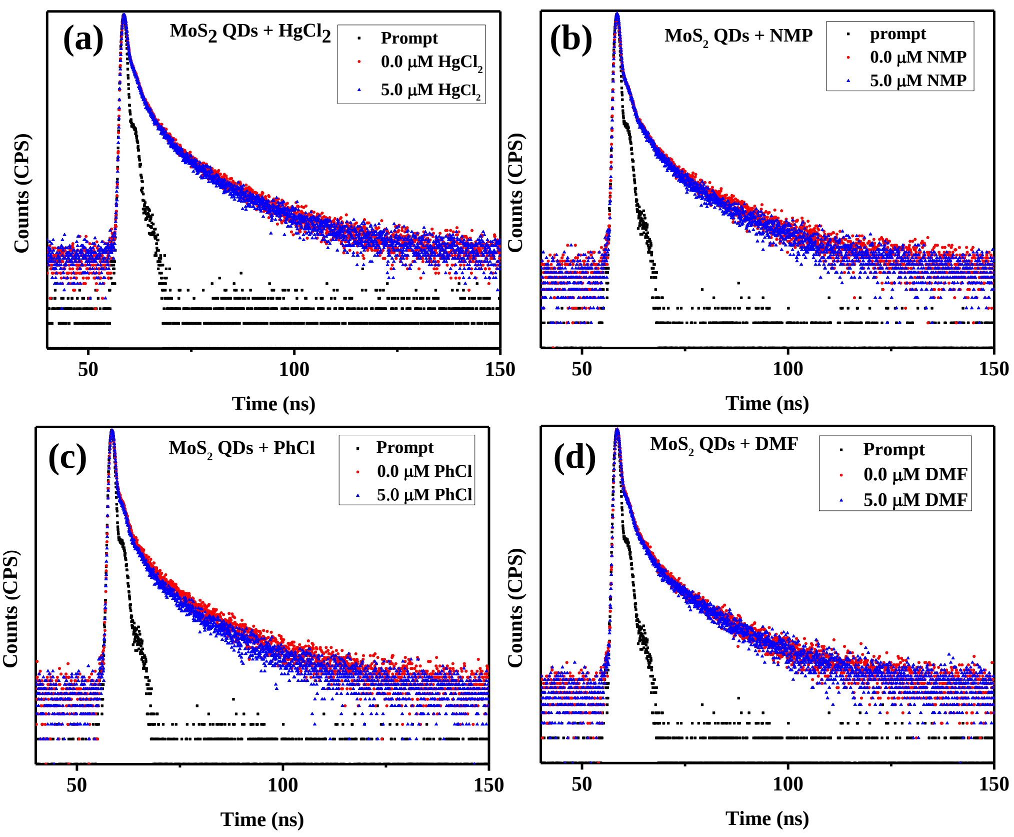

Fig. S4. Lifetime decay curve of MoS_2 QDs and MoS_2 QDs with different analytes such as (a) $HgCl_2$, (b) NMP, (c) PhCl, and (d) NMP. The instrument response is termed as prompt in the graphs.

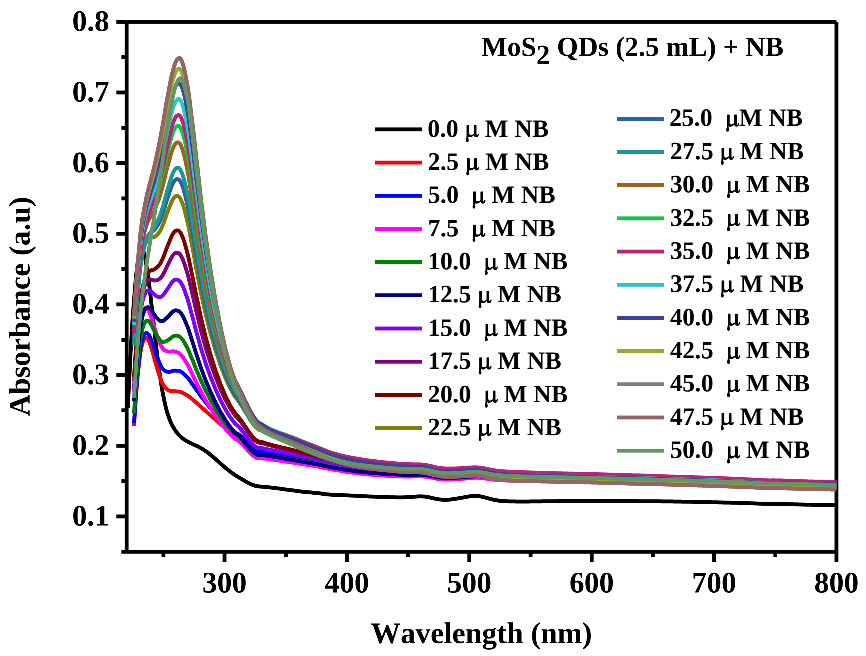

Fig. S5. UV-visible spectra of MoS_2 QDs (black) and MoS_2 QDs at different concentrations of NB (2.5μM-50 μM).

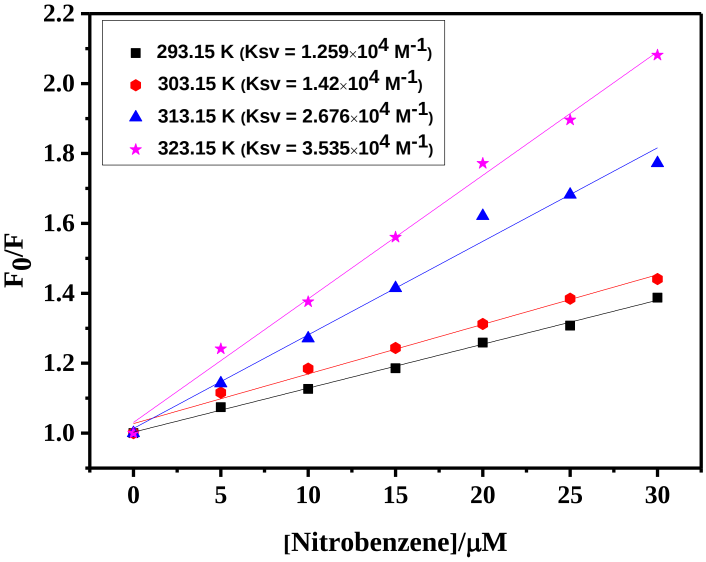

Fig. S6. Plots of F_0/F vs [Nitrobenzene] at different temperatures. The solid line shows fit to the simple Stern-Volmer equation (eq (1).

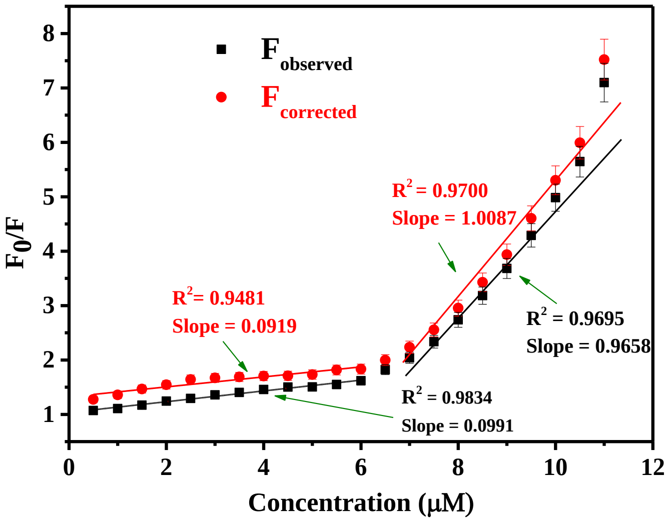

Fig. S7. Relationship between F_0/F vs concentration of NB of $F_{observed}$ and $F_{corrected}$, showing linear plot with different slopes. F_0 and F are the steady state intensity before and after the addition of NB, respectively.

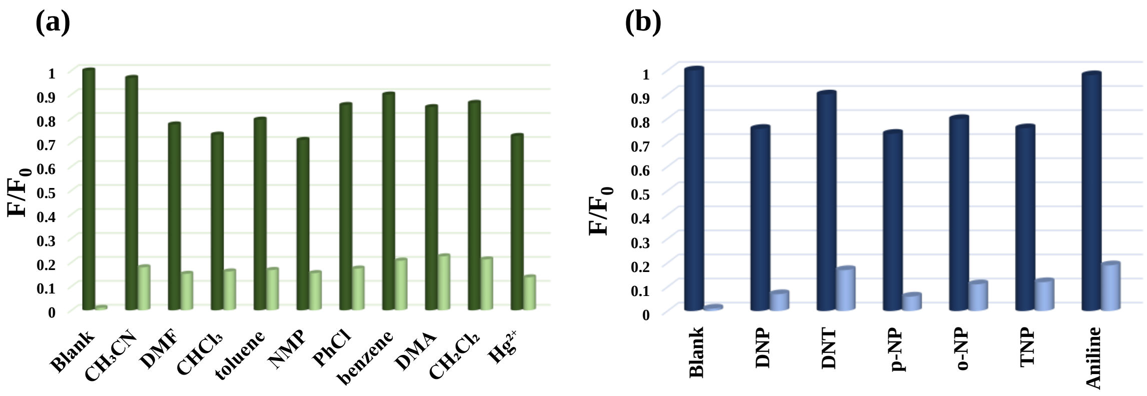

Fig. S8 (a) Percentage of quenching by different interfering analytes (0.01mM) before and after the addition of (0.01mM) NB. (b) Percentage of quenching by different interfering nitro explosives (0.01mM) before and after the addition of (0.01mM) NB.

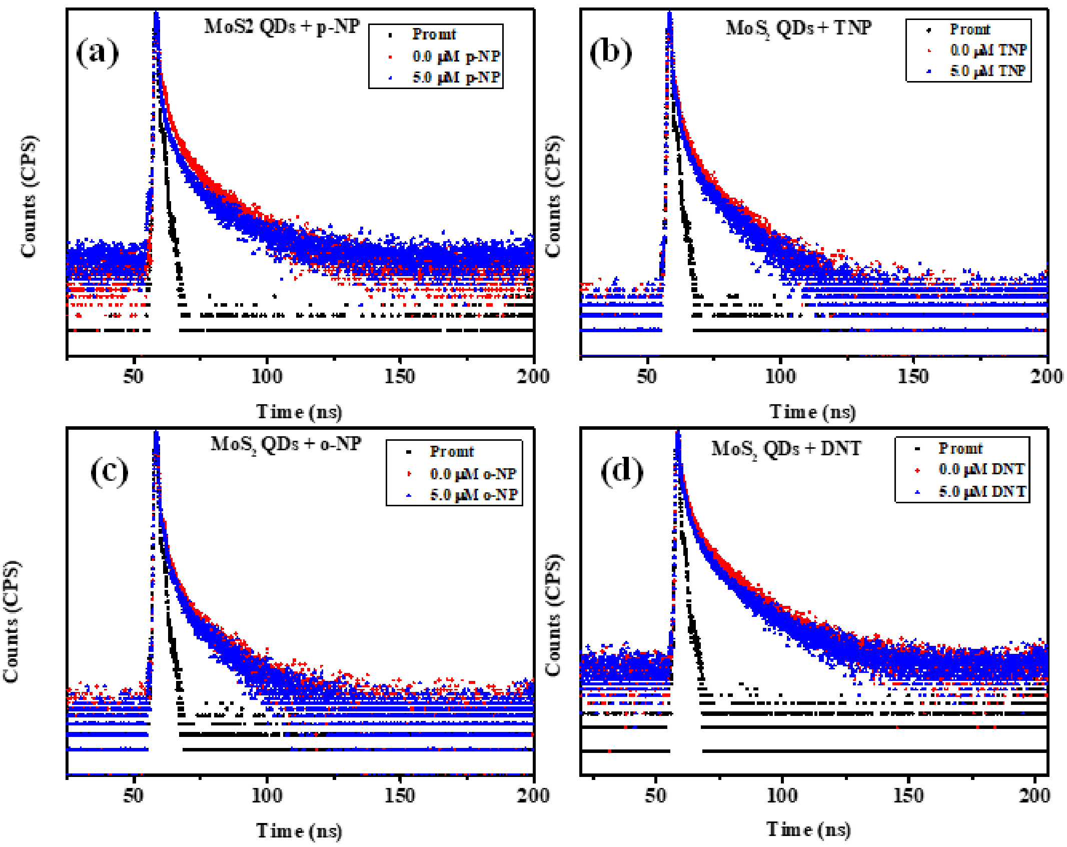

Fig. S9. Lifetime decay curve of MoS_2 QDs and MoS_2 QDs with different nitro explosives such as (a) p-NP, (b) TNP, (c) o-NP, and (d) DNT. . The instrument response is termed as prompt in the graphs.

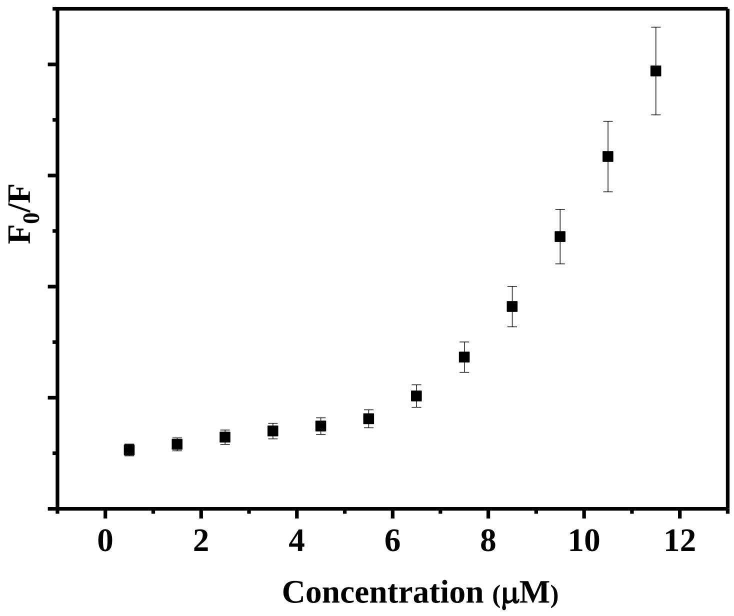

Fig. S10. This figure demonstrates the reproducibility of the sensor material. Each data point is derived from three different batches of MoS_2 QDs and three repeat measurements.

Materials	Mechanism	Linear range	Limit of detection	Selectivity	Reusability	Ref
Lanthanide-titanium oxo clusters (LTOCs)	Static quenching	0-9 ppm	10.5 ppb	PhMe, PhCl, PhCHO, $PhNH_2$	Not discussed	1
Metal-organic frameworks (MOFs)	Electron transfer	0-200 µM	28.9 µM	MeCN, DMF, THF, MeOH, benzene, toluene	Not discussed	2
Graphene oxide	Redox reaction	0.1-0.9 µM	66 nM	phenol, catechol, hydroquinone, resorcinol	Yes	3
Eu(III) doped zinc MOFs	Electron transfer	0-25 ppm	0.97 ppm	MeOH, IPA, EtOH, THF, DMA	Not discussed	4
ZnSnO3-g-C3N4	Electron transfer	30–100 µM	2.2 µM	4-nitrophenol, 1- chloro,2,4-dinitrobenzene, 1-bromo,2-nitrobenzene, 1-iodo,2-nitrobenzene	Yes	5
Chitin hydrogel stabilized graphite (GR-CHI)	Electron transfer	0.1 to 594.6 µM	37 nM	p-, o-, & m-nitrophenol, uric acid, ascorbic acid, dopamine.	Yes	6

Ag NPs	π–π interaction	0.5 to 900 µM	0.261 µM	4-bromo nitrobenzene, 4-chloro nitrobenzene, 4-nitroaniline, 4-nitrobenzoic acid, acetamido phenol	Yes	7
Au-NPs	Redox reaction	0.1 – 600 µM	0.016 µM	nitro anilines, 4-chloro nitrobenzene, phenol, resorcinol, catechol, hydroquinone, 4-nitro benzoic acid, 4-nitro phenol	Yes	8

Table S1: Comparison of present NB sensor with previously reported fluorescence based NB sensors.

System	τ_1 (ns)	α_1 (%)	τ_2 (ns)	α_2 (%)	τ_3 (ns)	α_3 (%)	<τ> (ns)	χ^2
MoS_2 QD	1.78	19.54	6.13	32.84	19.46	47.62	16.62	1.18
1 µM NB	1.75	19.85	5.75	30.97	16.37	49.88	14.05	1.19
2 µM NB	1.68	26.01	5.66	30.12	16.13	43.87	13.50	1.20
3 µM NB	1.54	26.12	5.41	29.34	15.83	44.55	13.36	1.20
4 µM NB	1.45	29.54	5.40	30.34	15.99	40.12	13.21	1.17
5 µM NB	1.25	30.36	4.38	37.55	15.19	45.09	12.80	1.17
6 µM NB	0.84	39.86	4.35	28.43	15.18	23.71	11.52	1.13
7 µM NB	0.76	50.3	4.11	26.71	14.97	22.98	11.44	1.2
8 µM NB	0.59	54.26	4.02	29.01	13.68	22.73	10.39	1.2
9 µM NB	0.51	57.69	3.94	28.63	12.14	23.68	9.29	1.2
10 µM NB	0.45	66.68	3.46	26.78	11.05	26.54	8.77	1.2

Table S2. The lifetime component of MoS_2 QDs and MoS_2 QDs-NB shows concentration dependence (of NB) on lifetime value. All decay profiles are fitted into tri-exponential functions. A decrease in average lifetime values of MoS_2 QDs-NB system implies the interaction of excited state MoS_2 QDs and with NB.

Fluorescence quenching efficiency calculation

Using steady-fluorescence data

$$E_F = 1-(F_{DA}/F_D) \qquad \text{----- (Eq. S1)}$$

Where,
F_{DA} is the fluorescence intensity of Doner in the prescence of Accepter.
F_D is the fluorescence intensity of Doner alone.
Fluorescence quenching efficiency = 79 %

Dynamic quenching efficiency calculation

Using time-resolved fluorescence data

$$E_D = 1-(\tau_{DA}/\tau_D) \qquad \text{------ (Eq. S2)}$$

Where,
τ_{DA} is the fluorescence lifetime of Doner in the presence of Accepter.
τ_D is the fluorescence lifetime of Doner alone
Dynamic quenching efficiency = 47.3 %

Reference

1 H. Zheng, Y. K. Deng, M. Y. Ye, Q. F. Xu, X. J. Kong, L. S. Long and L. S. Zheng, *Inorg. Chem.*, 2020, **59**, 12404–12409.

2 L. Yang, C. Lian, X. Li, Y. Han, L. Yang, T. Cai and C. Shao, *ACS Appl. Mater. Interfaces*, 2017, **9**, 17208–17217.

3 A. M. S. M. M. Otrokov, I. I. Klimovskikh, F. Calleja, J. H. D. O. Vilkov, A. G. Rybkin, D. Estyunin, S. Mu, H. O. A. L. Vázquez de Parga, R. Miranda and A. A. F. Guinea, J. I. Cerdá, E. V. Chulkov, 2018, 0–13.

4 S. Xian, H. L. Chen, W. L. Feng, X. Z. Yang, Y. Q. Wang and B. X. Li, *J. Solid State Chem.*, 2019, **280**, 120984.

5 S. Vinoth, P. Mary Rajaitha and A. Pandikumar, *Compos. Sci. Technol.*, 2020, **195**, 108192.

6 R. Sakthivel, S. Palanisamy, S. M. Chen, S. Ramaraj, V. Velusamy, P. Yi-Fan, J. M. Hall and S. K. Ramaraj, *J. Taiwan Inst. Chem. Eng.*, 2017, **80**, 663–668.

7 C. Karuppiah, K. Muthupandi, S. M. Chen, M. A. Ali, S. Palanisamy, A. Rajan, P. Prakash, F. M. A. Al-Hemaid and B. S. Lou, *RSC Adv.*, 2015, **5**, 31139–31146.

8 R. Emmanuel, C. Karuppiah, S. M. Chen, S. Palanisamy, S. Padmavathy and P. Prakash, *J. Hazard. Mater.*, 2014, **279**, 117–124.
